# Mindfulness based stress reduction study design of a longitudinal randomized controlled complementary intervention in women with breast cancer

**DOI:** 10.1186/1472-6882-13-248

**Published:** 2013-10-02

**Authors:** Elisabeth Kenne Sarenmalm, Lena B Mårtensson, Stig B Holmberg, Bengt A Andersson, Anders Odén, Ingrid Bergh

**Affiliations:** 1 Research and Development Centre, Skaraborg Hospital, Skövde, Sweden; 2 Institute of Health and Caring Sciences, Sahlgrenska Academy at Gothenburg University, Gothenburg, Sweden; 3 Palliative Research Centre, Ersta Sköndal University College and Ersta Hospital, Stockholm, Sweden; 4 School of Life Sciences, University of Skövde, Skövde, Sweden; 5 Department of Surgery, SU/Sahlgrenska University Hospital, Gothenburg, Sweden; 6 Microbiology and Immunology, Göteborg University, Göteborg, Sweden; 7 Department of Mathematical Sciences, Chalmers University of Technology, Gothenburg, Sweden

**Keywords:** Breast cancer, Mindfulness based stress reduction, Randomized controlled trials, Well-being

## Abstract

**Background:**

The stress of a breast cancer diagnosis and its treatment can produce a variety of psychosocial sequelae including impaired immune responses. Mindfulness Based Stress Reduction (MBSR) is a structured complementary program that incorporates meditation, yoga and mind-body exercises. Despite promising empirical evidence for the efficacy of MBSR, there is a need for randomized controlled trials (RCT). There is also a need for RCTs investigating the efficacy of psychosocial interventions on mood disorder and immune response in women with breast cancer. Therefore, the overall aim is to determine the efficacy of a Mindfulness Based Stress Reduction (MBSR) intervention on well-being and immune response in women with breast cancer.

**Methods and design:**

In this RCT, patients diagnosed with breast cancer, will consecutively be recruited to participate. Participants will be randomized into one of three groups: MBSR Intervention I (weekly group sessions + self-instructing program), MBSR Intervention II (self-instructing program), and Controls (non-MBSR). Data will be collected before start of intervention, and 3, 6, and 12 months and thereafter yearly up to 5 years. This study may contribute to evidence-based knowledge concerning the efficacy of MBSR to support patient empowerment to regain health in breast cancer disease.

**Discussion:**

The present study may contribute to evidence-based knowledge concerning the efficacy of mindfulness training to support patient empowerment to regain health in a breast cancer disease. If MBSR is effective for symptom relief and quality of life, the method will have significant clinical relevance that may generate standard of care for patients with breast cancer.

**Trial registration:**

ClinicalTrials.gov: NCT01591915

## Background

Breast cancer is one of the most feared diseases among women, and for many women a breast cancer diagnosis and its subsequent treatment are accompanied by dealing with a spectrum of physical and psychological challenges [1,2]. Women with breast cancer are at risk of psychological morbidity [3], and up to 30% of women diagnosed with breast cancer develop mood disorder, either anxiety or depressive symptoms, within one year of diagnosis [4,5]. While breast cancer is a major stressor for any woman, there is great variability in women’s emotional responses and their ability to mobilize the resources to cope with distress. However, although levels of distress tend to decrease over time, a subset of women with breast cancer remain highly distressed [6,7]. A growing area of interest is the personal growth and transformation of individuals as they adjust to a life-altering event and an increasing body of literature illustrates that many people experience significant personal growth despite severe trauma or disease [8,9]. Notwithstanding recurrent breast cancer, women who view their disease as a challenge and an opportunity for personal change are able to create wellness by being in the present moment [10].

The stress of a breast cancer diagnosis and its treatment can produce a variety of psychosocial sequelae including impaired immune responses [11]. An increasing body of research indicates that stress-related psychosocial factors are associated with higher cancer incidence and higher mortality [12]. Data suggest that psychological stress has a significant negative effect on cellular immune responses, such as lowered natural killer cells (NK cells) and T lymphocytes (T cells) [13]. Previous research indicates that NK and T cells are sensitive to different aspects of the stress response in women with breast cancer [14]. In addition, T cells have been linked to recurrence [15] and survival [16]. Other important parameters are cytokines, such as interleukin-6 (IL-6) and interleukin-8 (IL-8), which independently show correlations with breast cancer disease stage and progression [17-20].

The recognition of psychosocial needs in cancer patients has led to the development of psychosocial interventions, indicating a variety of positive effects on coping, quality of life, emotional and functional adjustment [21,22]. The literature on psychosocial interventions for cancer fails to meet criteria for establishing treatment efficacy and does not address issues of cost-effectiveness [23]. Although evidence suggests beneficial effects of psychosocial interventions [21,22], the physiological benefits for breast cancer patients remain unclear [24]. Furthermore, there are no existing large-scale studies prospectively investigating the efficacy of psychosocial intervention on coping and immune response in women with breast cancer [24].

Mindfulness Based Stress Reduction (MBSR) is a standardized program incorporating mindfulness meditation, yoga practices and other techniques designed to reduce suffering and improve health and well-being in patients with a wide range of chronic pain and stress disorders [25-27]. MBSR has also been shown to improve mood and reduce symptoms of stress in mixed groups of cancer patients [28]. The primary goal of MBSR is to develop the capacity to be aware in each moment, by “paying attention in a particular way: on purpose, in the present moment, and non-judgmentally” [25]. MBSR is a structured group-formatted 8-week course, attended once a week by patients for an average of 2 hours, together with daily practice of homework assignments. While noting the efficacy of MBSR on different patient populations, several reviews have pointed out the inherent methodological problems in the published studies [29,30]. Two systematic reviews of MBSR interventions for the treatment of anxiety and depression emphasize the methodological shortcomings which create uncertainties about the efficacy of treatment. The reviewed studies that reported a statistically significant reduction in anxiety and depression did not include control groups or follow-up data, and future studies with improved methodologies are needed to test the efficacy of the mindfulness component of the intervention [31,32].

The MBSR program was shown to have beneficial effects on immune function, reduced cortisol levels, improved Quality of Life (QoL), and increased coping effectiveness in a non-randomized group of women with breast cancer as compared with women who received the usual care [33]. A recent randomized study [34] showed that cancer patients who received a mindfulness intervention reported less perceived stress and posttraumatic avoidance symptoms and increased positive states of mind. This study indicates that the improvements in psychological well-being resulting from the MBSR intervention could have been explained in terms of increased levels of mindfulness. Other study results indicate a positive adjustment of cortisol levels as a result on MBSR program [35]. Evidence from uncontrolled studies of breast and prostate cancer patients also suggests that MBSR improved QoL and decreased stress symptoms [36], and altered cortisol and immune patterns [37]. Study results suggest that MBSR may influence the functioning of the hypothalamic-pituitary-adrenocortical (HPA) axis [35,38]. These pilot data from studies investigating the relationships between MBSR and hormone levels highlight the need for better-controlled studies in this area [38]. These results raise important questions as to whether mindfulness based interventions actually enhance mindfulness and whether such changes in mindfulness are related to positive outcome in the experience of health, strengthened immune system or disease-free survival.

Despite promising empirical evidence that the efficacy of MBSR improves mood and reduces symptoms of stress, there is a need for randomized, controlled studies including homogenous patient populations and using reproducible and stringent methodological procedures. In addition to self-reported psychosocial and functional measures of health and illness, assessment of biological markers are also needed in future mindfulness based interventions [30].

### Aim and outcome measurements

**Figure 1:**
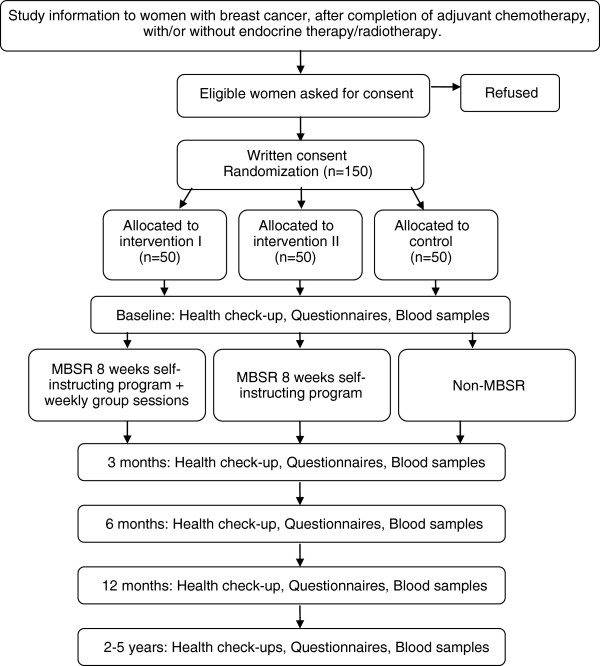
**Study design including enrollment, allocation and follow-up.** MBSR = Mindfulness Based Stress Reduction.

The overall aim is to determine the efficacy of a Mindfulness Based Stress Reduction (MBSR) intervention on well-being and immune response in women with breast cancer. This paper presents in-depth information on the design of the study (Figure 1). The study design follows the Consort recommendations [39-41].

#### Primary outcome

The efficacy of MBSR on mood disorders, evaluated in terms of anxiety and depression.

#### Secondary outcomes

The efficacy of MBSR on:

● coping capacity,

● symptom experience,

● quality of life,

● health status,

● personal growth,

● level of mindfulness,

● physiological response, disease progression, survival,

● analyses of utility and health economics, and

● analyses of the usability of MBSR in clinical care.

## Methods

### Design

Patients diagnosed with early stage breast cancer will be consecutively recruited from two surgical centers, to participate in this three-armed randomized controlled complementary intervention study. The study design is presented in Figure 1.

Written consent will be obtained before enrolment. Confidentiality will be assured, and participants will be advised that they are free to withdraw from the study at any time, and respect will be given to the participant’s condition. The study is registered in ClinicalTrials.gov: NCT01591915.

### Participants

In this randomized, controlled intervention study, patients diagnosed with breast cancer will be consecutively recruited to participate after completion of adjuvant chemotherapy, with/or without endocrine therapy.

Research nurses will contact eligible patients at the first follow-up appointment for patients receiving hormonal therapy or at the last treatment for patients undergoing chemotherapy. After oral and written information, interested patients will provide written consent to participate in the study. Participants who agree to participate will be invited to a first baseline health check-up appointment, including collection of blood samples, completion of questionnaires, a health check-up, and finally random assignment to one of three groups. Randomization will be conducted in blocks of 9, 12 and 15 blocks varied randomly. The assignment codes will be kept in sequentially numbered, opaque, sealed envelopes, prepared by the research team. At the time of allocation, the research nurse will pick the sequential envelope, write the participant’s name and personal registration number on the envelope, and then open it.

#### Inclusion criteria

Patients diagnosed with early-stage breast cancer:

receiving hormonal therapy, or:

after completion of adjuvant chemotherapy,

with or without radiotherapy

#### Exclusion criteria

Patients with other advanced illness at diagnosis, and/or:

ongoing major depression

ongoing Herceptin therapy

previously use of MBSR

### Training of instructors

Trained by a senior MBSR instructor, four registered nurses, skilled in patient learning, have been trained as certified MBSR instructors. Briefly, the instructor training consists of: 1 day of introduction to MBSR; 8 weeks of instructors’ own MBSR practice including 20 minutes sessions, 6 days/week; 2 days focused on practical training in group sessions; 8 week course in leading a group of patients who attend once a week for an average of 2 hours; 2 days focused on instructors’ experience of leading patient groups; and 1 day examination and certification.

### Intervention

It is well known that attention, expectations and good caring treatment contribute to the placebo effect. In order to minimize the impact of a situation with “a self-selected treatment and caring response to an enthusiastic therapist may be part of the active components of the alternative medical field” [42], two intervention groups and one control group will be included. All women receive standard care (SC) according to the national and local guidelines recommendations [43].

Participants will be randomized into one of three groups:

– SC and MBSR Intervention group I (8 weeks self-instructing program + instructor and weekly group sessions)

– SC and MBSR Intervention group II (8 weeks self-instructing program)

– SC and Non-MBSR (Controls)

Participants in MBSR Intervention group I and MBSR Intervention group II will participate in an 8-week tutorial of MBSR practice with homework assignments consisting of 20 minutes sessions, 6 days/week. Participants will be provided with information material including a 20-page introduction to mindfulness training, CD, diary and training program. Only participants in MBSR Intervention group I will take part in a structured group-formatted 8-week course that patients attend once a week for an average of 2 hours. Led by a certified MBSR instructor, these weekly group sessions will be focused on the participant’s experience of mindfulness, and include gentle yoga and meditation training. Owing to the differences between the two interventions groups, blinding will not be possible.

### Data collection

Socio-demographic data will be collected through chart review and interviews. Data characteristics are age, marital status, living situation, employment status, children, occupation and educational level. Clinical characteristics investigated are type of treatment, tumor characteristics, and co-morbidity. Patient self-reported response (questionnaires) and immune response (blood samples) will be collected, and health checks conducted, at baseline (before start of intervention), after the intervention for MBSR Intervention groups I and II, follow-up will be conducted one months after the intervention, and at similar time points of 3 months (non-MBSR), 6 months, 12 months, and thereafter yearly up to 5 years. Health checks including current health status and treatment, and blood samples will be performed by research nurses. Patient outcome will be collected by following the questionnaires, health check-ups and bio-markers.

### Measuring outcomes

#### Primary outcome

##### Mood disorder

The Hospital Anxiety and Depression Scale (HAD) is one of most widely used instruments to screen for anxiety and depression or psychosocial distress in cancer patients [44-46]. HAD is a 14-item questionnaire consisting of two sub-scales, anxiety and depression. Each response is rated on a four-point scale. Subscale scores range from 0 to 21. Scores of 0–7 indicate “normal”, 8–10 “borderline” and scores of >11 or more on either sub-scale are considered to represent risk of psychological morbidity [44-47]. The internal consistency of reliability for HAD Depression and HAD Anxiety are satisfactory, with Cronbach’s alpha 0.72 - 0.89, respectively 0.78 - 0.93 [46].

#### Secondary outcomes

##### Coping capacity

Personal coping resources are evaluated using the Sense of Coherence scale (SOC) [48,49]. The SOC short version scale consists of 13 items on a 7-point Likert scale. The SOC scale evaluates perceived comprehensibility (5 items), manageability (4 items) and meaningfulness (4 items). A higher score represents a stronger sense of coherence [50]. The reliability and validity of the SOC scale has been proven in many studies and different languages with a Cronbach’s α range of 0.74 to 0.93 [51-53].

##### Symptom experience

Symptoms (frequency, severity and distress) are measured using the Memorial Symptom Assessment Scale (MSAS), a multidimensional questionnaire consisting of 32 symptoms [54]. The MSAS generates the Total Symptom Burden Scale (TMSAS) and the Global Symptom Distress Index (GDI). The MSAS has been proven to be a reliable and valid multidimensional measure of symptom experience in cancer populations [54,55].

##### Health-related quality of life

Breast cancer specific HRQoL is evaluated using the 10 item visual analogue scale of the International Breast Cancer Study Group Quality of Life Core Questionnaire (IBCSG QoL). The questionnaire includes global indicators for physical well-being, mood, coping, social support and subjective health estimation, and indicators of symptoms covering possible specific disease and treatment-related effects. Higher scores indicate a higher level of symptoms/problems. The reliability and validity of the questionnaire has been established in several studies [56,57].

##### Health status

The SF-36 measures eight domains of health including physical functioning, role limitations due to physical health, bodily pain, general health perceptions, vitality, social functioning, role limitations due to emotional problems, and mental health [58]. The SF-36 yields a score for each of these eight domains, a total score for physical and mental health, and a global health utility index. Maximum score is 100 points. Higher scores indicate better health status. The reliability measurements of the SF-36 are consistently good [58-61].

##### Personal growth

Posttraumatic Growth Inventory (PTGI) is a measure of positive life changes after traumatic or life-altering events [62]. The PTGI consist of 21 items, rated on a 6-point Likert scale, divided into five dimensions: Relating to others (7 items), New possibilities (5 items), Personal strength (4 items), Spiritual change (2 items), and Appreciation of life (3 items). The PTGI yields a score for each of these subscales, and a total score. The PTGI has shown good reliability in previous research of breast cancer survivors, with Cronbach’s alpha for the PGI total score was 0.96 [63].

##### Mindfulness

Five Facets of Mindfulness Questionnaire (FFMQ short form). FFMQ developed by Baer and colleagues [64]. The FFMQ short form consists of 29 items, and yield five factors that appear to represent elements of mindfulness as it is currently conceptualized: Observing, Describing, Acting with awareness, Non-judging of inner experience and Non-reactivity to inner experience.

##### Blood sampling

NK cell function will be measured using a newly developed assay called flow cytometric assay of natural killer cell immune response in activated whole blood (FANKIA; 1). Briefly the cell line K562 is mixed with whole blood and the mix is centrifuged and incubated in 37°C for 2 hours. After staining with an antibody that recognizes K562, the number of remaining K562 cells is determined using flow cytometry and the lytic activity of the NK cells is calculated. Flow cytometry will be performed using a standard multicolour procedure to detect the main lymphocyte populations in human blood. Staining for regulatory T cells will also be performed. Cytokine analyses of IL-6 and IL-8 in serum will be performed using the automatic enzyme immunoassay system Immulite 1000 according to the standard procedure from the manufacturer.

##### Health check-ups

Research nurses will conduct health check-ups including blood pressure and heart rate, at baseline and follow-ups. A protocol for baseline socio- demographics and medical characteristics, and follow-up protocols for current medical conditions, including disease progression, will be used to report health status.

##### Analyses of utility and health economics and usability of MBSR

The utility of MBSR in clinical settings, and health economic impact and costs will be described in relation to sick leave, health care patterns, drug costs and mortality.

### Sample size calculation

To detect significant differences between three groups (MBSR Intervention I and II, and non-MBSR): 50 participants per group (150 participants in total) are needed in order to achieve a statistical power of 80%. The power calculation was based on alpha of 0.05 and expected mean effect size difference between the groups (difference in change between baseline and 3 months follow-up) being at least 1 unit at HAD-scale.

### Statistical analyses

Descriptive statistics will be used to summarize socio-demographic and clinical characteristics. Pearson’s correlation coefficient will be calculated to determine the strength of relationships between selected variables. Univariate and multivariate analyses of variance for repeated measures (ANOVA/MANOVA) will be used to compare between groups. For comparison of skewed variables, a non-parametric test will be used. Regression analyses will be used to identify predictors. In multivariable stepwise procedures only variables that provide statistically significant contributions will be included in the analyses (p < 0.05).

### Interviews

With the purpose of exploring how participants experience the effects of MBSR on mood and HRQoL, interviews will be conducted. A further purpose, from a patient perspective, is to examine whether MBSR is clinically relevant as a complementary method in breast cancer. With the purpose of describing the process over time, repeated interviews will be conducted during and after the intervention. Grounded theory is a qualitative method tailored to exploring processes, actions and meaning. It is a systematic method of comparative analysis and a strategic method of generating theory grounded in data [65,66].

## Quality aspects

The following competences are represented in the research group: Research manager Elisabeth Kenne Sarenmalm, RN, PhD, Adjunct Lecturer, Research and Development Center, Skaraborg Hospital, Skövde, Institute of Health and Caring Sciences, Sahlgrenska Academy at University of Gothenburg, experienced in research in breast cancer patients, expert in psycho-oncology, and a certified MBSR instructor. Lena B Mårtensson, RNM, PhD, Associate Professor, Schools of Life Sciences, University of Skövde, and expert in randomized controlled trials in complementary methods. Stig B Holmberg, specialist in tumor surgery, MD, Associate Professor, Breast Department, SU/Sahlgrenska University Hospital, Gothenburg, medical advisor, expert in international, randomized, controlled breast cancer trials, and patient recruitment. Bengt A Andersson, specialist in clinical immunology, MD, Associate Professor, Immunological Laboratory, SU/Sahlgrenska University Hospital, Gothenburg, clinical immunological advisor and expert in stress and immune response. Anders Odén, Professor in Biostatistics, at Chalmers University of Technology, Gothenburg, statistical advisor and expert in statistical methods, biostatistics, statistical aspects of epidemiology. Ingrid Bergh, RN, PhD, Professor, Schools of Life Sciences, University of Skövde, and expert in patient-reported outcomes assessment.

The senior MBSR instructor will continue as a supervisor during the intervention in order to support MBSR instructors and establish internal validity. A research controller, responsible for logistics and protocol monitoring, and coordinating follow-ups, provide guarantee for sustainability. Four research nurses, responsible for inclusion, randomization and health check-ups, is a guarantee for continuity during follow-up. Finally, continuous monitoring will ensure that the intervention is implemented according to protocol, and with inclusion and exclusion of participants follow-up to avoid selection bias. Exclusion of patients, including reason for exclusion, will be reported in a reject log. Process evaluations will be conducted using intermittent checks in order to assure that the intervention procedures are performed correctly and follow the study protocol.

## Ethical considerations

In studies including patients diagnosed with a serious illness such as breast cancer, ethical problems may arise. Fundamental ethical principles including respect for individuals, and not causing harm will be observed. The risk of privacy intrusion is minimized by requiring informed consent. Participation in the study is voluntary and can always be discontinued if the patient so desires. Responsiveness and flexibility about the patient’s condition and motivation for participating in the study will considered. Ethical approval has been granted by the Etichal Research Committee, University of Göteborg D:nr 499–9; 12/11/2009. Approval has also been granted by the medical directors of the participating hospitals.

All patient data from the study are confidential and no unauthorized person will have access to this information. In data processing, name and personal identity number will be replaced by a code so that no individual can be identified. Only the principal investigator of the study will have access to the code key. In published study reports, individuals will not be identified. Handling personal data in Sweden is governed by the Personal Data Act [67]. All data will be computer processed and stored for at least 10 years.

The blood samples will be stored encrypted, and will not be directly traceable back to individuals. The samples, as well as a corresponding identification list (code key), will be stored safely and separately. Samples may be used only in ways to which the participants consented. They can only be made available to a new research project after participants submit a new agreement and/or approval is granted by the Ethical Research Committee. The participant has the right to request, without explanation, that samples be destroyed or made anonymous according to the Swedish act “Biobanks in Medical Care Act” [68].

## Discussion

Women with breast cancer have unique rehabilitation needs. As the most common invasive cancer disease among women worldwide, a high frequency of both physical and psychological problems follows breast cancer diagnosis and treatment. The major goal of rehabilitation is to help each patient to achieve maximal function within the limitations imposed by the disease and its subsequent treatment.

Beyond the physical challenges of the disease and its subsequent treatment, breast cancer patients experience a variety of emotional reactions and psychological symptoms, which may have a significant impact on quality of life. Symptom relief and health promotion are central in the care of women with breast cancer. The present study may contribute to evidence-based knowledge concerning the efficacy of mindfulness training to support patient empowerment to regain health in a breast cancer disease. If MBSR is effective for symptom relief and quality of life, the method will have significant clinical relevance that may generate standard of care for patients with breast cancer. If so, the method will also be of great benefit to other patient groups. There is a substantial body of research on disease and treatment of severe illness, but very few studies directly examine factors that promote health. MBSR may promote health by engaging and strengthening an individual’s internal resources for optimizing recovery from illness, and it may also improve the ability to cope with mood disturbance and symptoms of stress.

## Competing interests

The authors declared that they have no competing interest.

## Authors’ contributions

EKS conceived and conducts this study. EKS, LM and IB contributed to the conception and design of the study. SH provides feedback in each phase of the study. BA carries out the cytometric assays and the immunoassays. AO participated in the design and is responsible for statistical analyses. All authors read and approved the final manuscript.

## Pre-publication history

The pre-publication history for this paper can be accessed here:

http://www.biomedcentral.com/1472-6882/13/248/prepub
